# Baroreflex Activation Therapy in Patients With Resistant Hypertension: A Single‐Center Real‐World Analysis of Blood Pressure Response and Procedural Safety

**DOI:** 10.1111/jch.70337

**Published:** 2026-11-09

**Authors:** Shaima Al‐asbahi, Olaf Oldenburg, Henrik Fox

**Affiliations:** ^1^ Kardiologie Faculty of Medicine Ruhr‐Universität Bochum Bochum Germany; ^2^ Department of Cardiology Ludgerus‐Kliniken Münster GmbH, Clemenshospital Münster Germany; ^3^ Heart Failure Department Herz‐ und Diabeteszentrum Nordrhein‐Westfalen, Universitätsklinik Der Ruhr‐Universität Bochum Bad Oeynhausen Germany

## Abstract

Baroreflex activation therapy (BAT) has emerged as a treatment option for patients with resistant hypertension; however, real‐world data on blood pressure response and procedural safety of the second‐generation Barostim Neo system remain limited.

In this single‐center retrospective study, 37 patients with resistant hypertension who underwent BAT implantation between 2013 and 2024 were analyzed. Changes in systolic office blood pressure (OBP) and 24 h ambulatory blood pressure (ABP) were assessed. Patients were classified as responders if any reduction in systolic blood pressure from baseline was observed. Periprocedural safety and procedural characteristics were also evaluated.

In the overall cohort, no statistically significant reduction in systolic blood pressure was observed (ABP: 159 ± 27 to 157 ± 24 mmHg; OBP: 172 ± 19 to 165 ± 29 mmHg; both *p* > 0.05). However, a substantial proportion of patients showed a clinically meaningful response. Among responders, systolic ABP decreased significantly from 170 ± 24 to 141 ± 15 mmHg and systolic OBP from 178 ± 16 to 146 ± 17 mmHg (both *p* < 0.0001). A combined improvement in ABP and OBP was observed in 48% of patients with complete data. The mean implantation time was 74 min, and periprocedural complications occurred in 13.5% of cases. Generator replacement procedures were shorter and associated with a low rate of transient complications.

In this real‐world cohort, BAT using the Barostim Neo system appears to be a safe therapeutic option, although not all patients benefit. Among responders, BAT is associated with substantial and clinically relevant blood pressure reduction. Further prospective studies are needed to improve patient selection and evaluate long‐term outcomes.

## 1 Introduction

Arterial hypertension remains one of the major global challenges in modern medicine. In the year 2000, an estimated 26.4% of the adult population worldwide was affected, and the prevalence was projected to rise to 29.2% by 2025 [1].

Hypertension is recognized as one of the most significant risk factors for the development of coronary artery disease, cerebrovascular stroke, heart failure, peripheral arterial disease, vision loss, chronic kidney disease, and premature death [2].

Despite the availability of numerous antihypertensive medications, a substantial proportion of patients fail to achieve adequate blood pressure control under pharmacologic therapy.

Resistant hypertension represents a particularly severe and treatment‐refractory form of the disease. It is defined as the failure to reach target blood pressure levels (<140/90 mmHg in most patients or <130/80 mmHg in those with diabetes mellitus or chronic kidney disease) despite treatment with at least three antihypertensive agents at maximally tolerated doses, including a diuretic.

Although the exact prevalence of resistant hypertension remains uncertain, it is estimated to affect 20%–30% of all hypertensive patients [3].

Resistant hypertension is frequently associated with multiple comorbidities, including obstructive sleep apnea, diabetes mellitus, chronic kidney disease, and atherosclerotic vascular disease [3]. Patients with resistant hypertension have an approximately 2.9‐fold higher risk of experiencing fatal or non‐fatal cardiovascular events compared to responders [4].

It is well established that patients with resistant hypertension exhibit increased sympathetic nervous system activity relative to parasympathetic tone [5].

As early as the 1930s, attempts were made to reduce vasoconstrictive sympathetic effects through surgical sympathectomy, involving resection of sympathetic ganglia. Although this approach achieved significant reductions in blood pressure, it was ultimately abandoned due to severe adverse effects such as orthostatic hypotension, impotence, and urinary incontinence [6, 7].

Experimental studies in the 1990s and early 2000s demonstrated that electrical stimulation of baroreceptors in animal models (rats and dogs) led to a significant and sustained reduction in arterial blood pressure [8, 9, 10].

Building on these findings, baroreflex activation therapy (BAT)—also known as barostimulation—was developed for clinical application in humans in the mid‐2000s.

The therapeutic effect of BAT is mediated through electrical stimulation of baroreceptors in the carotid sinus, leading to reduced sympathetic outflow and enhanced parasympathetic activity, which together lower systemic vascular resistance and arterial blood pressure.

The first‐generation device, known as the Rheos system (CVRx Inc., Minneapolis, MN, USA), consisted of a pulse generator (volume 43.4 cm^3^) implanted in the pectoral region, connected to two bilateral carotid sinus leads. Each lead ended in four finger‐like electrodes encircling the carotid sinus bulb, requiring surgical exposure of both carotid bifurcations for placement [11].

In a large, double‐blind, randomized, placebo‐controlled trial (Rheos Pivotal Trial), the Rheos system demonstrated a significant and sustained reduction in blood pressure among patients with resistant hypertension. However, due to the complex surgical procedure, the trial reported a nearly 30% rate of procedure‐related complications [12].

These findings prompted the further refinement of the technology.

In the early 2010s, the second‐generation Barostim Neo system was introduced. This device features a smaller pulse generator (<40 cm^3^) and a single carotid sinus lead with one contact electrode that is sutured directly onto the carotid sinus [11].

Observational studies have reported sustained reductions in systolic blood pressure and good tolerability of the Barostim Neo system, supporting its potential role in the management of resistant hypertension.

Although no randomized controlled trials have yet evaluated the antihypertensive efficacy of the Barostim Neo system, initial clinical experiences suggest a promising blood pressure‐lowering effect.

Despite these encouraging results, data on the perioperative safety, procedure duration, and real‐world outcomes of Barostim Neo implantation remain limited.

Therefore, the present study aims to provide a retrospective analysis of patients treated with Barostim therapy for resistant hypertension, focusing on procedural characteristics, complications, and blood pressure outcomes in a single care center over an 11‐year period.

## 2 Methods

### 2.1 Study Design

This study is a single‐center, retrospective analysis of patients treated at Ludgerus Klinikum, Clemenshospital Münster (Germany), who underwent implantation of a Barostim Neo system (CVRx Inc., Minneapolis, MN, USA) between 2013 and 2024.

The primary aim was to evaluate the effect of BAT on mean systolic 24 h ambulatory blood pressure (ABP) and office systolic blood pressure.

The study included all eligible patients who underwent BAT implantation during the study period; no a priori sample size calculation was performed due to the retrospective nature of the analysis.

Patient data were obtained from paper‐based and electronic medical records. The study was conducted in accordance with the Declaration of Helsinki and approved by the Ethics Committee of the Medical Association Westfalen‐Lippe (reference 2024‐396‐f‐S).

A total of 37 patients who underwent BAT implantation between 2013 and 2024 were included. All procedures were performed by the hospital's surgical team using second‐generation devices(Barostim Neo system (CVRx Inc., Minneapolis, MN, USA))

In 86% (32/37), implantation was right‐sided. Follow‐up was coordinated by the Department of Internal Medicine and Cardiology at 6 weeks, 3 months, and every 6 months thereafter.

Patients were asked to provide 24 h ABP recordings at each visit, and office blood pressure (OBP) was measured and documented. Device parameters (amplitude, frequency, pulse width) and/or antihypertensive medication were adjusted based on BP response and tolerance.

Patients were classified as responders if there was any reduction in systolic blood pressure compared to baseline was achieved at least at one of the two follow‐up visits.

### 2.2 Inclusion and Exclusion Criteria

Patients were eligible if they underwent BAT implantation at the Clemenshospital between 2013 and 2024 and had ≥1 documented follow‐up ≥3 months post‐implantation.

Anatomical eligibility for BAT implantation required at least one carotid bifurcation located below the mandibular angle, free of atherosclerotic plaques causing >50% luminal stenosis of the distal common carotid artery or the internal carotid artery.

Patients were excluded if the implantation was performed at an external hospital with follow‐up at the Clemenshospital or vice versa, or if medical records were incomplete or follow‐up data were missing.

### 2.3 Study Endpoints

The primary endpoint was the change in systolic 24 h ABP from baseline to follow‐up.

Secondary endpoints included changes in office systolic blood pressure, and periprocedural complications.

Periprocedural complications were defined as any adverse event occurring during implantation or hospitalization related to the procedure or device.

### 2.4 Data Collection

Baseline characteristics (demographics, comorbidities, antihypertensive therapy, BP values) were extracted from clinical records. Periprocedural parameters (procedure duration, intraoperative findings, complications) were obtained from operative reports and discharge summaries.

Two follow‐up time points were defined, corresponding to documented routine clinical follow‐ups starting at 3 months after implantation. Only follow‐ups with complete documentation of the required data were included in the analysis.

To assess the potential impact of medication adjustments on blood pressure outcomes, changes in antihypertensive medication burden were additionally evaluated in patients with complete ABP follow‐up data.

### 2.5 Device Description

The Barostim Neo system comprises a subcutaneously implanted pulse generator in the pectoral region and a unilateral carotid sinus lead delivering electrical stimulation to carotid baroreceptors.

### 2.6 Statistical Analysis

Continuous variables are presented as mean ± standard deviation (SD) or median (interquartile range, IQR), as appropriate. Categorical variables are presented as counts and percentages.

Changes between baseline and follow‐up were assessed using the paired *t*‐test.

*p*‐value <0.05 was considered statistically significant.

All analyses were performed using Microsoft Excel.

Missing data were handled by excluding incomplete follow‐up visits rather than entire cases. If a follow‐up visit lacked the required data, the next available visit with complete documentation was used for analysis. Given the retrospective design and limited sample size, multiple imputation was not performed.

Given the exploratory nature of this retrospective study, no formal adjustment for multiple comparisons was performed. Therefore, all p‐values should be interpreted as exploratory.

## 3 Results

### 3.1 Baseline Characteristics

A total of 37 patients who underwent BAT implantation between 2013 and 2024 at the Clemenshospital Münster and completed regular clinical follow‐up were included in the analysis.

The mean age was 73 ± 13 years, 57% (*n* = 21) were male, and the mean body mass index (BMI) was 30 ± 5 kg/m^2^.

Comorbidities were common: 18 patients (49%) had diabetes mellitus, 15 (41%) had coronary artery disease, and 17 (46%) had peripheral or cerebrovascular arterial disease.

Evidence of hypertensive target organ damage included a history of stroke in 6 patients (16%), myocardial infarction in 5 patients (14%), and chronic kidney disease (≥ stage 3) in 10 patients (27%).

At baseline, patients were treated with a median of 5 antihypertensive medications (interquartile range [IQR] 3–7), including a diuretic in all cases.

6 patients (16%) had undergone renal denervation at least three months prior.

Before BAT implantation, the mean systolic OBP was 171 ± 19 mmHg, and the mean systolic 24 h ABP was 160 ± 27 mmHg. Baseline demographic and clinical characteristics of the study population are summarized in Table 1.

**TABLE 1:** Baseline characteristics of the study population.

Variable	Value
Number of patients, *n*	37
Age (years), mean ± SD	73 ± 13
Male sex, *n* (%)	21 (57%)
Body‐mass index (kg/m^2^), mean ± SD	30 ± 5
Diabetes mellitus, *n* (%)	18 (49%)
Coronary artery disease, *n* (%)	15 (41%)
Peripheral/cerebrovascular arterial disease, *n* (%)	17 (46%)
History of stroke, *n* (%)	6 (16%)
History of myocardial infarction, *n* (%)	5 (14%)
Chronic kidney disease stage ≥ 3, *n* (%)	10 (27%)
Antihypertensive medications, median (IQR)	5 (3‐7)
Prior renal denervation, *n* (%)	6 (16 %)
Baseline systolic office BP (mmHg), mean ± SD	171 ± 19
Baseline systolic ambulatory BP (mmHg), mean ± SD	160 ± 27

Abbreviations: BP, Blood pressure; IQR, Interquartile range.

Complete ABP documentation at baseline and two follow‐up visits ≥3 months was available for 28 patients.

Complete OBP records were available for 35 patients, and 27 patients had fully documented values for both parameters. The patient selection process and allocation to endpoint‐specific analysis cohorts are illustrated in Figure 1.

**FIGURE 1:**
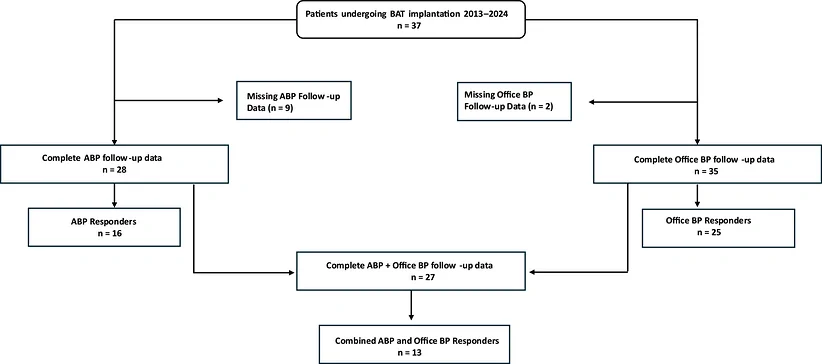
Patient inclusion and allocation to endpoint‐specific analysis cohorts.

### 3.2 Blood Pressure Outcomes

In the subgroup with complete ABP data (*n* = 28), systolic blood pressure decreased from 159 ± 27 mmHg to 157 ± 24 mmHg (*p* > 0.05).

In 12 of these 28 patients, no reduction in blood pressure was observed.

Among ABP‐responders(*n* = 16), representing 57% (16/28) of this subgroup, systolic ABP decreased significantly from 170 ± 24 mmHg to 141 ± 15 mmHg (*p* < 0.0001).

In patients with complete OBP documentation (*n* = 35), a reduction trend from 172 ± 19 mmHg to 165 ± 29 mmHg was observed (*p* > 0.05).

Among OBP responders (*n* = 25) (71%, 25/35), systolic OBP decreased significantly from 178 ± 16 mmHg to 146 ± 17 mmHg (*p* < 0.0001).

Among the 27 patients with complete ABP and OBP data, 48% (13/27) demonstrated a simultaneous improvement in both parameters.

In this subgroup, significant reductions were observed:

- ABP: 176 ± 28 mmHg → 147 ± 23 mmHg (*p* = 0.0001)
- OBP: 180 ± 19 mmHg → 146 ± 22 mmHg (*p* = 0.0004)

Detailed changes in systolic ambulatory and OBP are presented in Table 2.

**TABLE 2:** Changes in systolic blood pressure in ABPM and OBPM after barostim therapy.

	Complete ABP Data	Complete OBP Data	Complete ABP + OBP Data
Number of patients (*n*)	28	35	27
Systolic BP before BAT (mmHg)	159	172	ABP/OBP: 169/159
Systolic BP after BAT (mmHg)	157	165	ABP/OBP: 165/156
Responders (*n*)	16	25	13
Responders (%)	57	71	48
Responders systolic BP before BAT (mmHg)	170	178	ABP/OBP: 176/180
Responders systolic BP after BAT (mmHg)	141	146	ABP/OBP: 147/146
Systolic BP reduction in responders (mmHg)	29	32	ABP/OBP: 29/34
Systolic BP reduction in responders (%)	17	18	ABP/OBP: 16/19

Male patients were less likely to achieve a combined therapeutic response than female patients (38% vs. 57%).

Exploratory subgroup comparisons did not reveal any obvious differences in baseline demographic or clinical characteristics between responders and non‐responders. However, formal multivariable analyses were not performed because of the limited sample size. Figure 2 illustrates systolic blood pressure values before and after BAT in the overall cohort and responder groups.

**FIGURE 2:**
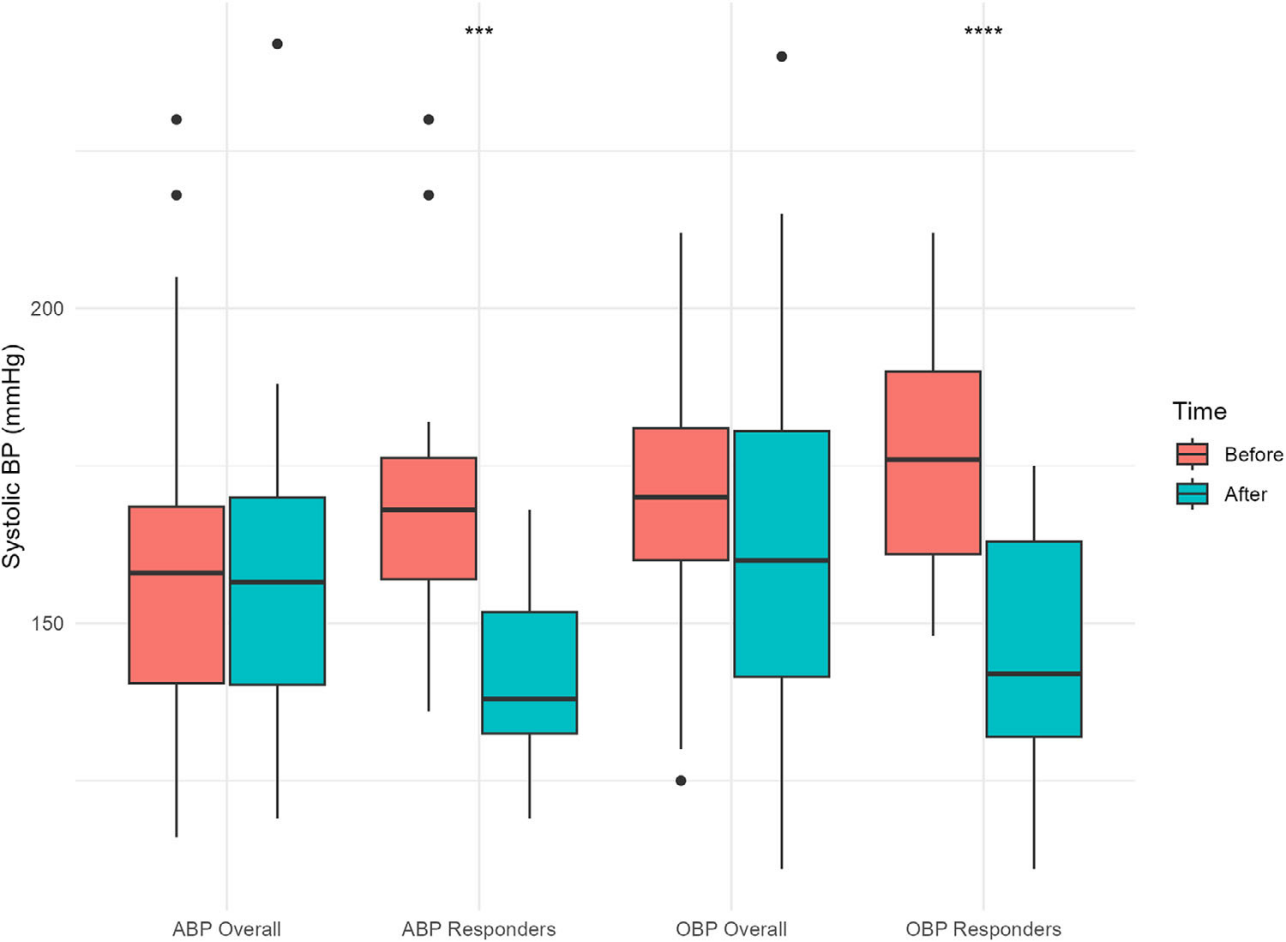
Systolic blood pressure before and after barostim therapy in total and responder groups.

### 3.3 Changes in Antihypertensive Medication Burden

To evaluate the potential impact of medication adjustments on blood pressure outcomes, changes in antihypertensive medication burden were analyzed in patients with complete ABP follow‐up data (*n* = 28). The median number of antihypertensive medications remained unchanged at five medications before and after BAT. Medication burden was reduced in 14 patients (50%), remained unchanged in 13 patients (46%), and increased in only one patient (4%).

### 3.4 Sensitivity Analysis

To assess the robustness of the responder definition, an additional sensitivity analysis was performed using a clinically meaningful threshold of ≥10 mmHg reduction in systolic blood pressure. Using this criterion, 11 of 28 patients (39%) were classified as responders based on ABP measurements, whereas 20 of 35 patients (57%) met the criterion based on OBP measurements. These findings indicate that a substantial proportion of patients achieved clinically meaningful blood pressure reductions following BAT.

### 3.5 Safety Outcomes

The mean implantation time was 74 min (range: 40–120 min). Battery longevity until generator replacement averaged 4 ± 1,25 years.

Generator replacement procedures lasted a mean of 27 min (range: 5–122 min).

Periprocedural complications during initial implantation occurred in 13.5% (5/37) of cases, including:

- Twiddler's syndrome requiring revision
- Transient facial nerve palsy due to intraoperative mandibular nerve compression
- External jugular vein injury controlled surgically
- Defective lead requiring revision
- Lead revision due to a mismatch between patient anatomy and lead length.

A total of 33 generator replacement procedures were performed during the study period. Complications occurred in 9.1% (3/33) of procedures and consisted exclusively of transient neurological events, including two cases of motor aphasia and one transient ischemic attack. All neurological symptoms resolved completely during follow‐up without permanent sequelae.

## 4 Discussion

In this single‐center retrospective analysis, BAT using the second‐generation Barostim Neo system demonstrated a favorable safety profile and clinically meaningful reductions in both office and ambulatory systolic blood pressure in a substantial proportion of patients with resistant hypertension. Despite the advanced age and high comorbidity burden of the study population, more than half of the patients experienced a significant therapeutic response.

The antihypertensive effect observed in our cohort aligns with results from previous observational studies investigating Barostim Therapy. Both the DEBuT‐HT [13, 14] and BAT‐HTN trials [15] reported significant reductions in systolic blood pressure, particularly in responders, and demonstrated an acceptable safety profile. Early registry data also suggest sustained long‐term benefit and improved cardiovascular risk markers following BAT [16].

Although randomized controlled evidence remains limited, these convergent real‐world findings support BAT as an important therapeutic option for carefully selected patients with resistant hypertension.

Of note, approximately 48% of patients with complete ambulatory and OBP documentation experienced a simultaneous improvement in both measures. This observation underscores the clinical robustness of BAT, as improvement in only office measurements may reflect a white‐coat effect rather than a true reduction in cardiovascular risk. The high response rate in this severely affected population suggests that BAT effectively targets sympathetic overactivity, a key pathogenic driver in resistant hypertension.

Importantly, the observed treatment effect remained evident when a more stringent responder definition was applied. In a sensitivity analysis using a threshold of ≥10 mmHg systolic blood pressure reduction, 39% of patients achieved a clinically meaningful reduction in ABP and 57% achieved a clinically meaningful reduction in OBP. These findings support the robustness and clinical relevance of the observed antihypertensive effect.

Male patients appeared less likely to achieve a combined therapeutic response than female patients. However, the present study was not adequately powered to identify independent predictors of treatment response. Given the limited sample size, formal multivariable analyses were not performed. Larger prospective studies are needed to identify clinical predictors of BAT response and optimize patient selection.

To evaluate the potential influence of medication adjustments on treatment outcomes, changes in antihypertensive medication burden were additionally analyzed in patients with complete ABP follow‐up data. The median number of antihypertensive medications remained unchanged at five medications before and after BAT. Medication burden was reduced in 14 patients (50%), remained unchanged in 13 patients (46%), and increased in only one patient (4%). These findings suggest that the observed blood pressure reductions are unlikely to be explained solely by intensification of pharmacological therapy.

The observed perioperative complication rate of 13.5% should be interpreted in the context of the vascular surgical nature of BAT implantation and the advanced age and high comorbidity burden of the study population. Although this rate was lower than the approximately 30% complication rate reported for the first‐generation Rheos system, clinically relevant complications still occurred.

Notably, the observed complications included one case of Twiddler syndrome and two lead‐related revision procedures. While these events did not result in permanent sequelae, they highlight the importance of meticulous surgical technique, appropriate device fixation, and careful patient follow‐up. Similar device‐ and lead‐related complications have also been reported in patients undergoing conventional cardiac implantable electronic device procedures, including pacemaker implantation [17].

Overall, the findings suggest that the second‐generation Barostim Neo system is associated with improved procedural safety compared with earlier BAT systems. Nevertheless, procedure‐related complications remain an important consideration in clinical practice and should be carefully balanced against the potential antihypertensive benefits of BAT.

A total of 33 generator replacement procedures were performed during the study period, with transient neurological events occurring in three procedures (9.1%). Although generator replacement does not involve direct manipulation of the carotid bifurcation or stimulation lead, several potential mechanisms may explain these events. Given the advanced age and substantial cardiovascular comorbidity burden of the study population, transient neurological symptoms may have been related to perioperative blood pressure instability, acute hemodynamic changes during procedural stress, thromboembolic events, anesthesia‐related factors, or pre‐existing cerebrovascular disease. Sudden increases or decreases in blood pressure during the perioperative period may have contributed to transient cerebral hypoperfusion or cerebrovascular events in susceptible patients.

Importantly, all neurological deficits resolved completely without permanent sequelae. Nevertheless, these findings highlight the need for careful perioperative monitoring and blood pressure management during generator replacement procedures in this high‐risk patient population.

### 4.1 Study Limitations

Several limitations must be acknowledged. First, this was a retrospective, non‐randomized study with a relatively small sample size, limiting the generalizability of the findings.

In addition, the relatively small sample size limited the statistical power to detect subgroup differences and identify potential predictors of treatment response.

Furthermore, the absence of a sham‐control or medical therapy control group precludes establishing a causal relationship between BAT and the observed blood pressure reductions.

Second, complete ABP follow‐up data were available for only 28 of 37 patients. Missing data were handled by excluding incomplete follow‐up visits, which may have introduced selection bias and affected the robustness of the primary endpoint analysis. Given the retrospective design and limited sample size, multiple imputation was not considered appropriate.

Third, medication adjustments during follow‐up, although clinically necessary, may have contributed to blood pressure changes independent of BAT.

Finally, the follow‐up duration varied among patients, precluding a standardized long‐term outcome evaluation.

In addition, no formal adjustment for multiple comparisons was performed. Consequently, the reported *p*‐values should be interpreted in an exploratory rather than confirmatory context.

### 4.2 Clinical Implications and Future Directions

Despite these limitations, the present study provides further evidence that BAT offers a therapeutic benefit in a real‐world cohort of patients with resistant hypertension who have exhausted conventional pharmacologic and interventional treatment options. Future prospective, randomized trials with standardized follow‐up and biomarker‐based patient stratification are needed to confirm these findings and refine candidate selection

## 5 Conclusion

In this single‐center retrospective study of patients with resistant hypertension treated with the second‐generation Barostim Neo system, BAT was associated with meaningful reductions in systolic office and ABP in a substantial proportion of patients, despite advanced age and a high burden of comorbidities. Approximately half of the patients with complete ambulatory and OBP data achieved a combined response, and responders showed marked systolic blood pressure reductions.

Procedural risk was acceptable, with short implantation and generator replacement times and a manageable rate of procedure‐related complications, all of which resolved without permanent sequelae. These findings support BAT as a feasible and safe adjunctive treatment option for carefully selected patients with resistant hypertension in routine clinical practice.

However, given the retrospective design, limited sample size, missing follow‐up data, lack of a control group, and heterogeneous follow‐up duration, the results should be interpreted as hypothesis‐generating. Prospective, randomized trials with standardized follow‐up and predefined response criteria are needed to better define the role of Barostim therapy in the management of resistant hypertension and to identify predictors of treatment response.

## Funding

The authors have nothing to report.

## Ethics Statement

The study was approved by the local ethics committee of the Medical Association Westfalen‐Lippe(reference 2024‐396‐f‐S).

## Consent

Due to the retrospective nature of the study, patient consent was waived.

## Conflicts of Interest

The authors declare no conflicts of interest.

## Data Availability

The data that support the findings of this study are available from the corresponding author upon reasonable request.
